# Role of *Helicobacters* in Neuropsychiatric Disease: A Systematic Review in Idiopathic Parkinsonism

**DOI:** 10.3390/jcm9072159

**Published:** 2020-07-08

**Authors:** Rosalind M. Tucker, Aisha D. Augustin, Bu’ Hussain Hayee, Ingvar Bjarnason, David Taylor, Clive Weller, André Charlett, Sylvia M Dobbs, R John Dobbs

**Affiliations:** 1 Pharmaceutical Sciences, King’s College, London SE1 9NH, UK; Rosalind.Tucker@kcl.ac.uk (R.M.T.); Aisha.Augustin@kcl.ac.uk (A.D.A.); David.Taylor@slam.nhs.uk (D.T.); Clive.Weller@kcl.ac.uk (C.W.); Andre.Charlett@phe.gov.uk (A.C.); John.Dobbs@kcl.ac.uk (R.J.D.); 2 The Maudsley Hospital, London SE5 8AZ, UK; 3 Gastroenterology, King’s College Hospital, London SE5 9RS, UK; B.Hayee@nhs.net (B.H.H.); IngvarBjarnason@mac.com (I.B.); 4 Statistics, Modelling and Economics, National Infection Service, Public Health England, London NW9 5EQ, UK

**Keywords:** *Helicobacter pylori*, virulence markers, non-*Helicobacter pylori Helicobacters*, eradication, Parkinson’s disease, aetiopathogenesis, brady/hypokinesia, rigidity

## Abstract

Interest in an aetiopathogenic role for *Helicobacter* in neuropsychiatric diseases started with idiopathic parkinsonism (IP), where the cardinal signs can be assessed objectively. This systematic review, using an EMBASE database search, addresses Oxford Centre for Evidence-Based Medicine based questions on the inter-relationship of *Helicobacter* and IP, the benefits of eradicating *Helicobacter* in IP and the outcome of not treating. The search strategy was based on Preferred Reporting Items for Systematic Reviews and Meta-Analyses guidelines: 21 of 204 articles met the inclusion criteria. The results show that the assumption that any benefit of *Helicobacter* eradication results from improved levodopa bioavailability is unjustified. The inter-relationship between *Helicobacter* and IP is well-established. *H. pylori* virulence markers (associated with autoimmunity and immune tolerance) influence the risk, severity and progression of IP. The birth cohort effect for virulence marker antibodies, seen in controls, is obliterated in IP, suggesting causality. Successful *H. pylori* eradication in IP is disease-modifying (even in anti-parkinsonian treatment-naïve patients) but not preventive. Hypokinesia regresses with eradication and overall motor severity lessens. Eradication may influence gastrointestinal microbiota adversely, unlocking the next stage in the natural history, the development of rigidity. Failed eradication worsens hypokinesia, as does the presence/persistence of *H. pylori* at molecular level only. Adequate prognostic assessment of the consequences of not treating *Helicobacter*, for IP, is prevented by a short follow-up. We conclude that *Helicobacter* is a pathophysiological driver of IP.

## 1. Introduction

*Helicobacter pylori* is one of the most common human pathogens, estimated to infect approximately 4.4 billion people worldwide, 45–80% of whom remain asymptomatic [1,2]. The principal site of infection is the gastric mucosal surface, where the organism is protected from acid by mucus and the generation of ammonia by urease [3]. Warren and Marshall first isolated these curved bacilli from patients with chronic gastritis (1983) and with peptic ulcer (1984) [4,5]. *Helicobacter pylori* is now the established primary cause of chronic gastritis, peptic ulcers, non-cardia gastric carcinoma and gastric mucosa-associated lymphoid tissue (MALT) lymphoma. Treating infection almost halves the incidence of non-cardia carcinoma [6]. Eradication is the treatment of choice for MALT lymphoma when confined to the stomach or with peri-gastric lymph node involvement [7]. Colonisation has also been reported in the mouth and liver [8,9,10]. The significance of *H. pylori* extends beyond the stomach [11,12,13,14,15,16,17,18,19,20]. Some conditions (iron and B12 deficiencies) are direct consequences of gastric infection and subsequent atrophy. Others appear to be truly extra-gastric. For example, *H. pylori* eradication has a well-established therapeutic effect on idiopathic thrombocytopenic purpura [16]. Candidature in Sjögren’s syndrome, atherosclerosis, migraine and rosacea rests largely on association [17,18,21].

It is widely recognised that *H. pylori* infection, especially strains with the pathogenicity marker cytotoxin-associated gene product (CagA), is associated inversely with Barratt’s oesophagus [22]. There are other interesting negative relationships, in keeping with the maintenance of immune homeostasis by *H. pylori* infection [23], in chronic immune-mediated disorders such as asthma, rheumatoid arthritis and inflammatory bowel disease [24]. Moreover, the treatment of *H. pylori* appears to increase the incidence of inflammatory bowel disease. A registry database in Taiwan showed that, in peptic ulcer disease, *H. pylori* eradication carried an increased risk of developing autoimmunity or inflammatory bowel disease compared with no eradication (assumed to represent no *H. pylori* infection since most would have been tested) [24].

What is the evidence for *Helicobacter* having a role in neuropsychiatric disease? From 2000, we identified, using the EMBASE database, 70 relevant papers linking neuropsychiatric diseases (idiopathic parkinsonism (IP), Alzheimer’s disease, mild cognitive impairment, affect disorders, schizophrenia/psychosis) to *Helicobacter*. Prior to the discovery of *Helicobacter pylori-*associated gastritis, an excess of concurrent (1961) and previously documented (1965) peptic ulcers was observed in IP [25,26]. Szabo proposed that an infectious agent was involved in both (1979) [27], before the name of that agent could simply be added [28]. Dopaminergic agonists can prevent duodenal ulcer relapse [29] but it is unknown whether this is due to *Helicobacter* suppression. The epidemiological fit of *H. pylori* with IP includes ubiquity, insidiousness, familial aggregation, immunological manifestations, linkage with drinking water sources [30] and associations with rosacea and migraine [17,18,31,32,33]. Most *H. pylori* infections are transmitted within a closely shared environment, from parent or sibling to infant [34], and then they persist. Indeed, in 1999, Charlett et al. reported that both IP patients and their siblings were three times more likely than controls to be seropositive for the *H. pylori* anti-urease antibody and that siblings were quantifiably “down-the-way” towards IP on objective measures of its facets [35]. Our aim here is to examine systematically the subsequent evidence defining the role of *Helicobacter* in IP.

## 2. Methods

### 2.1. Search Strategy

This systematic review was conducted and reported in line with the “Preferred Reporting Items for Systematic Reviews and Meta-Analyses” (PRISMA) guidelines [36]. The search strategy was based on the “Population, Intervention, Comparison, Outcome” (PICO) framework: P = people with or without IP; I = anti-*Helicobacter* treatment course; C = comparison of IP severity/manifestations pre- and post-*Helicobacter* eradication, and by *Helicobacter*-status according to IP-status or *vice versa*; O = severity/manifestations or status frequency.

The literature search was conducted using the Ovid EMBASE online database, with restriction to papers published between 2000 and 2019 in peer-reviewed scientific journals in, or with translation to, English. A systematic search combined two groups of keywords, one for the microbial insult (*Helicobacter* infection, *Helicobacter* (genus) or *Helicobacter pylori*) and the other for the IP target (Parkinson’s, Parkinson disease, idiopathic parkinsonism). We adopt the term “idiopathic parkinsonism” to refer to the target syndrome, with its variable combination of defined signs, in the absence of a recognised cause. Included were randomised or open *Helicobacter* eradication trials and cross-sectional observational (cohort or case–control) studies. Excluded from the systematic review (but not from the framing narrative reviews) were case reports, reviews and meta-analyses.

### 2.2. Subjective and Objective Measures of Idiopathic Parkinsonism

Outcomes for IP included global scores with particular reference to motor severity (e.g., Unified Parkinson’s Disease Rating Scale (UPDRS) Part III-motor section [37], Webster scale) [38]); disability Parkinson’s Disease Questionnaire (PDQ39) [39]; functional staging (Hoehn and Yahr) [40]; independence in activities of daily living (Schwab and England) [41]; or change in maintenance requirements for symptomatic treatment of IP. Regarding the clinical manifestations of IP, reference was made to motor subscores in UPRDS-III, but preference was given to the objective assessment of individual cardinal signs on a continuous scale (for example, mean stride length at free walking speed is a measure of hypokinesia, with high sensitivity to and specificity for IP [42]). Immune manifestations were included in the outcomes of note. We also included motor complications of levodopa therapy (UPDRS-IV) [37] and levodopa pharmacokinetics, since the effects of *Helicobacter* eradication might be ascribed to changes in levodopa bioavailability.

Outcomes with respect to the microbial insult included evidence of current status (from gastric biopsy, urea breath test (UBT), stool antigen) or past/concurrent status (eradication history, serology). Quantification of the antibody titre or pathogenicity marker profile was used where available.

### 2.3. Study Selection

**Figure 1:**
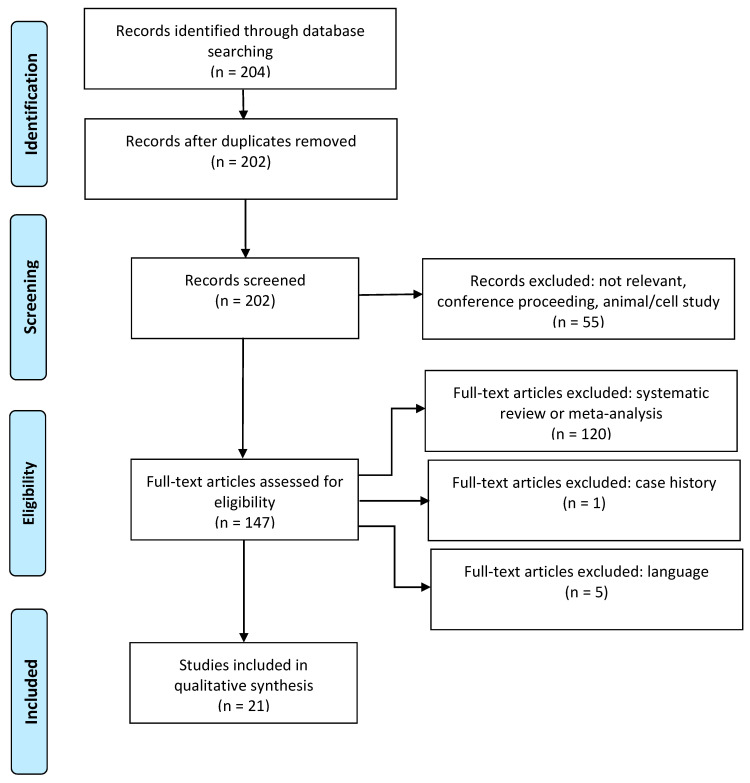
Preferred reporting for systematic review and meta-analysis (PRISMA) flow chart.

Figure 1 summarises the article selection process from search results, through screening and compliance with eligibility criteria, to inclusion. Two reviewers (R.M.T. and A.D.A.) scanned the search results independently. First, the titles and abstracts of the search results were checked, and then the full texts of possibly relevant articles were checked. After each step, the reviewers held a consensus meeting to discuss discrepancies in the selection. A third reviewer (R.J.D.) assessed any ambiguous selections.

### 2.4. Data Extraction and Questions Addressed

The following information was extracted from each included article (Tables 1–5): (i) citation; (ii) type of study: eradication trial, randomised (active eradication versus an alternative treatment such as placebo) or open, or cross-sectional comparison without or with retrospective/prospective data; (iii) cohort size; (iv) methodology for *Helicobacter* ascertainment and any confirmation of its eradication; (v) clinical outcome(s); and (vi) country where the study was performed.

The Oxford Centre for Evidence-Based Medicine (OCEBM) questions [43] that were addressed using the cumulative stratified evidence were as follows: (i) Does this intervention help (treatment benefits)?, (ii) What will happen if we do not add a therapy (prognosis)?, (iii) How common is the problem?

### 2.5. Risk of Bias

The methodological quality of each included study was assessed by two assessors (R.M.T., A.D.A), followed by a consensus meeting and a consultation with a senior researcher (R.J.D.) for overview. The Cochrane risk of bias assessment tool was applied to the randomised controlled trials [44].

## 3. Results

### 3.1. Search Results

The search returned 204 research titles (Figure 1). Excluded were two as duplicates, 18 as irrelevant, 33 as conference abstracts, four as animal studies, 120 as reviews or metanalysis, one as a case history and five on language. The remaining 21 original articles met the inclusion criteria, one being published as interim [45] and final [46] study reports. Studies were from Europe (11/20), East (4/20) and South East (3/20) Asia and the Middle East (2/20).

### 3.2. Study Characteristics 

Table 1 and Table 2 summarise the characteristics of the six trials on the effect of *Helicobacter* eradication on the global severity of IP or its cardinal signs.

Table 1 gives the two randomized controlled trials (RCTs) [45,46,47]. In both, active treatment was a 7-day course of triple therapy with two antimicrobials and a proton pump inhibitor. Both studies used endoscopic biopsy diagnosis of *H. pylori* infection. In the first [45,46], the choice of antimicrobials took into account in vitro sensitivities, whereas a fixed regimen was used in the second [47]. Only the former used molecular microbiology to detect/exclude low infection load. The comparator arm in the first trial [45,46] received placebo, dummy tablets without active ingredient being manufactured for the antimicrobials exhibited and for the proton pump inhibitor. The comparator in the second [47] was a 15-day course of allopurinol (as an antioxidant), with a placebo balance aimed at giving the same number of tablets per day in both treatment arms. In the first trial [45,46], use of the short (0.75–1.5 h) elimination, half-time (t½), anti-parkinsonian drug, levodopa, was an exclusion, in order to avoid confounding by drug-induced fluctuations. In the second [47], in contrast, having motor fluctuations on levodopa monotherapy was an inclusion criterion.

Recruitment in the first trial [45] was halted when marked deterioration in hypokinesia was noted with failed eradication. Despite this, the size of effect at 1 year, on intention-to-treat analysis, was more than 1.5 times that used in the original sample size calculation. The final report [46] allowed the inclusion of participants who had not reached de-blinding at the time of the interim analysis and follow-up for 2 years after the blinded phase. This included cross-over to open-active eradication in those with persistent *H. pylori* infection after placebo. Protocol analysis of proven eradication refines the contrast. In the second trial [47], there was no sample size calculation to achieve a pre-determined size of effect, but the sample size was slightly larger than in the first RCT. Follow-up was for 3 months [47]. Additional contrast of failed with successful eradication was made in both studies.

Table 2 shows four non-randomised trials. One had fixed-order cross-over from placebo to active eradication, with follow-up for 2 weeks in each phase [48]. Three were open eradication trials, with follow-up for 3 months [49,50] or 1 year [51]. Three used isotope-labelled urea breath tests (UBT) [49,50,51], the other serology. In the first two open trials, motor fluctuations attributed to levodopa were an inclusion criterion [48,49]. All participants were receiving levodopa in the third [50], some in the fourth [51].

Table 3 includes information from two of the above [49,50] plus six additional studies [52,53,54,55,56,57] which provide cross-sectional observational analyses of the effect of *Helicobacter*-status on the severity of IP and/or its cardinal signs. Half tested for current infection by UBT [49,50,55] or stool antigen [53], the rest used serology. Seven studies included people taking levodopa [49,50,52,53,54,55,56], motor fluctuations being an inclusion criterion in two [53,56]. There was no information on levodopa in the eighth study [57].

Table 4 lists studies of the effect of *Helicobacter*-status on outcomes other than global motor severity of IP and measurement of its facets [58,59,60,61].

Table 5 includes information from five of the above [52,54,56,57,60] plus four additional studies [62,63,64,65] on the comparative incidence or frequency of *Helicobacter* according to IP-status or, conversely, of IP according to *Helicobacter*-status. Two studies use analysis of National Registry data. One [63] defines populations by IP-status (tracing retrospectively for a *Helicobacter* eradication course), the other [65] by *Helicobacter*-status (tracing prospectively for IP medication). Two studies [52,62] address the obliteration of birth cohort effects (*i.e.* the increasing frequency of *Helicobacter*-seropositivity with age) in diagnosed IP. Four studies [54,56,57,60] report the relative frequency of *Helicobacter* in IP (by serology in 3, UBT in one) compared with a group of controls. In one study [60] using UBT, the controls were IP patients’ spouses, ignoring the possibility of *Helicobacter* transmission in the home environment. One study [64] addressed the frequency of non-*Helicobacter pylori Helicobacter* (NHPH) infection (detected by PCR on endoscopic biopsy) relative to that of *H. pylori*, according to IP-status. Lack of IP-status information in the large control group should have militated against finding a difference in frequency.

### 3.3. Study Findings

#### 3.3.1. Trials of *Helicobacter* Eradication in IP 

##### Randomised Controlled Trials of Effect on IP Severity or Facets

In the first [46] of the two RCTs summarised in Table 1, the protocol analysis of within-subject time trends in 6-weekly observations showed an improvement (by, on average, 7.3 cm) in mean stride length over the year after blinded active *Helicobacter* eradication therapy, compared with after placebo. The size of this effect was unaffected by whether participants were receiving stable long t½ anti-parkinsonian medication or were treatment-naïve. There was a marked worsening in stride length (29 cm) in the four participants with failed eradication, compared with the successful participants (even though persistence could be detected only at molecular level in two). This was reflected in changes in the global visual analogue ratings of stance and gait videos. The improvement in hypokinesia occurred despite an increase in rigidity post-eradication. The differential effect on hypokinesia and rigidity was replicated following open active, post-placebo. Improvements in hypokinesia plateaued from one year after active until the 3-year study endpoint. Rigidity plateaued in year 2 but showed a small deterioration in year 3.

In the second RCT [47], 3 months after the allocated treatment, all those receiving eradication therapy had an improved within-day grand total for multiple repeats of the UPDRS motor score, except for two with eradication failure. A lack of change or worsening was seen in the allopurinol comparator arm. “On-time” (defined there as the total duration with a 20% reduction in total UPDRS motor score from the day’s baseline) reflected the above findings.

##### Non-Randomised Trials of Effect on IP Severity or Facets

Of the four open trials (Table 2), one [50] found large improvements in the total UPDRS motor score and in PDQ-39, including its mobility domain, at 12 weeks post-eradication. Another [51] found an improvement, over the baseline, at one year following *Helicobacter* eradication, in both the total UPDRS motor score and its subscores relating to upper and lower limb brady/hypokinesia (after excluding two eradication failures). This improvement was over and above any longitudinal changes in a comparator IP group with untreated *Helicobacter* infections. 

Of the two trials with motor fluctuations as an inclusion criterion, in one [48], the UPDRS motor score, 2 h after levodopa, was better at 2 weeks after active eradication than it had been after the preceding placebo course. In the other [49], there was a numerical, but not statistically significant, improvement in the total UPDRS motor score 3 months after intervention (after excluding one eradication failure).

#### 3.3.2. Biological Gradients of *Helicobacter* Serology on Disease Burden in IP

The first study [52], in Table 3, is an observational study with dramatic effects. It describes the biological gradients, of clinically relevant size, between disease burden and a discriminant index for IP, based on the Western blot pattern of IgG antibodies against electrophoretically separated *H. pylori* antigens. The higher the index, the shorter the stride length and the greater global IP severity on the Webster scale. Moreover, the higher the index, the greater the deterioration in stride length over 4 years.

#### 3.3.3. Cross-Sectional Associations of *Helicobacter*-Status in IP 

##### With Global and Facet Assessments

Of the other studies in Table 3, *Helicobacter* positivity was associated with significantly worse total UPDRS motor scores [50,55,56], PDQ-39 scores [50,56] and bradykinesia in the timed walking and pegboard tests [55]. All of these studies, apart from one [56], assessed current *Helicobacter-*status. In contrast, two studies [53,57] showed no difference in total UPDRS motor scores between the *Helicobacter-*positive and -negative (in stool antigen and serology, respectively). One study [54] conflated *Helicobacter* seropositivity with a wider infection burden.

Functional staging (Hoehn and Yahr) was used as an outcome in several studies but was insensitive to *Helicobacter*-status across the board.

##### With Other Outcomes

Of the four cross-section observational studies in Table 4, the outcomes were body mass index [58], serum autoantibodies [59] and motor complications of levodopa therapy [60,61]. The latter are discussed below (Section 3.3.4). Regarding body mass index, although the odds of being underweight nearly tripled in the presence of seropositivity for the *Helicobacter* pathogenicity marker, vacuolating toxin (VacA), this was independent of IP-status [58]. The presence of “elevated autoantibodies against proteins essential for normal neurological functions” was greater in *H. pylori*-seropositive IP probands than in the seronegative IP probands [59].

#### 3.3.4. Effect of *Helicobacter* Eradication or Status on Clinical Pharmacology of Levodopa

##### Pharmacokinetics

*Helicobacter* eradication has been associated with an increased area under the 4-h levodopa concentration/time profile (related to a single morning dose) and the 11-h profile (which included subsequent levodopa dosing), both in a pilot study [48] and a subsequent RCT [47]. Ascertainment of *H. pylori*-status was by serology and endoscopic biopsy, respectively. However, in a cross-sectional study, current *H. pylori*-status did not significantly influence either the area under the 4-h concentration/time curve for levodopa or 3-O-methyldopa (its long t½ metabolite), with or without computed extrapolation beyond 4 h [53].

##### Pharmacodynamics

All three options for motor complications of levodopa therapy, decreased, increased and unaffected, have been reported as being associated with *Helicobacter*, mostly decreased. Following eradication, a shortened onset time of the levodopa effect, a prolonged switched-on-time and a tempering of end-of-dose wearing-off have been reported [47,49,50]. In cross-sectional studies, longer onset accompanied by shorter on-time distinguished the UBT-positive [49] and the seropositive [56] from the negative. On/off fluctuations were more apparent when UBT-positivity co-existed with small intestinal bacterial overgrowth (SIBO) [60]. In contradiction, another cross-sectional comparison showed less wearing-off with UBT-positivity in IP [61]. Others found no difference in dyskinesias or motor fluctuations between seropositive and seronegative groups [57].

#### 3.3.5. Longitudinal Follow-Up of Untreated *Helicobacter* Infection in IP

One study [51] had a cohort of 12 UBT-positive IP patients who chose not to receive *Helicobacter* eradication. On follow-up to one year, a differential effect of *Helicobacter* infection on UPDRS motor subscores was seen: rigidity became considerably better numerically (by two on a five-point scale), whilst upper and lower limb brady/hypokinesia and rest tremor became worse numerically. However, the placebo arm in a year-long blinded phase of an RCT of *H. pylori* eradication in IP [46] showed no change in either objectively measured brady/hypokinesia or rigidity, or in visual analogue ratings of hand tremor (whilst seated or during stance and walking).

In an inception cohort [65], with the ascertainment of *Helicobacter* infection by biopsy or UBT, the increased risk of IP diagnosis 4 years on was irrespective of eradication therapy.

#### 3.3.6. Inter-Relationship between *Helicobacter* and IP

##### How Common Is IP with or after *Helicobacter* Infection?

The increase in IP diagnosis with or after *Helicobacter* infection has been demonstrated in two studies [63,65] (Table 5). Interrogating the nationwide Danish registers showed that prior (5 years or more) prescription of *Helicobacter* eradication drugs was associated with a 45% increase in the diagnosis of IP [63]. The use of Taiwan’s National Health Insurance Register, where *Helicobacter* infection was primarily diagnosed by endoscopic mucosal biopsy, showed a 129% higher risk of IP associated with *Helicobacter* infection [65]. 

##### How Common Is *Helicobacter* in IP?

Of three cross-sectional comparisons of the frequency of testing seropositive for *Helicobacter* in people with and without IP (Table 5), two found a significant positive association [51,53] and one a negative association [54]. The lower frequency of seropositivity in IP in the latter may, in part, be attributable to excluding those with a history of *Helicobacter* eradication therapy. Another comparison found no difference in the frequency of UBT positivity, but controls were the spouses of the IP probands [60]. Non-*H. pylori Helicobacter* infection appears to be more common in IP: the relative frequency of *H. suis* (detected by PCR on gastric biopsies) to *H. pylori* was 10-times greater in IP patients than in routine gastroenterology patients undergoing endoscopic biopsy [64].

##### Effect of Age on Relationship between *Helicobacter* and IP

In Table 5, one study [62] shows that seropositivity for anti-urease-IgG antibody was greater in IP than in controls, until the age of 72 years. This was in contrast to the expected birth cohort effect (increasing frequency with age) seen in controls of similar social class representation. The predicted probability of being labelled as having IP was greatest with a particular profile of *H. pylori* virulence marker antibodies [52]. With this profile, the odds for parkinsonism increased from around aged 70 years, being fivefold by the age of 80 years.

### 3.4. Risk of Bias

Table 6 gives the available information on the risk of bias in the two double-blind RCTs of *Helicobacter* eradication [45,46,47]. Both studies appear of low risk generally. However, in one [47], there was a lack of clarity in the allocation concealment of treatments and in reporting whether the analysis was of proven eradication. There was also a risk of detection bias due to the repeated subjective assessments, with the potential for a carry-over effect.

## 4. Discussion

Our primary interest is in whether *Helicobacter* infection has an aetiopathogenic role in the evolution of IP and, if so, what processes are involved. A foundation is provided by understanding the inter-relationship of *Helicobacter* and IP and the effect of *Helicobacter* eradication, compared with no attempt at eradication or failed eradication. However, in a syndrome with a long prodrome and very variable manifestations between probands, where facets do not progress in parallel, unification under a single driver is unlikely [66]. Halting one disease progression pathway by eradicating *Helicobacter* may allow escape down subordinate ones. Moreover, there may be effect modifiers from the onset (e.g., human genetics) and *en route* (smoking).

There are three systematic reviews and/or meta-analyses of *Helicobacter* in IP. The 2011 review of six studies up to 2006 states the precondition that “potential benefits of *Helicobacter* eradication need to be balanced against the costs of screening and treatment” [67]. The worsening of hypokinesia with failed eradication was dismissed as an adverse treatment effect rather than being put together with improved hypokinesia with successful eradication. The 2017 meta-analysis of eight studies up to 2015 demonstrated a positive association between IP and *Helicobacter* [68]. The 2018 meta-analysis of 10 studies up to 2017 confirmed this association and found a global severity score for IP to be higher in the infected (seven studies), reduced after *Helicobacter* eradication (five) [69]. We review 20 studies up to 2019, addressing the following questions.

### 4.1. Three Questions Addressed by Systematic Review

#### 4.1.1. Effect of *Helicobacter* Eradication Therapy in IP

This systematic review complements the 2018 meta-analysis [69] in upgrading the level of OCEBM evidence to 1 for “Does this intervention (*Helicobacter* eradication) help?” The upgrade takes on board Level-2 evidence from RCTs [45,46,47] and an observational study with dramatic effect [52]. Three of the four non-randomised trials [48,50,51] (one of which had a dramatic effect [50]) provide supportive Level-3 evidence. In the other, the improvement seen did not reach statistical significance [49].

The “help” was manifested as a lessening of global motor severity and/or a reduction in brady/hypokinesia. A benefit over a short period could be a non-specific effect of eliminating infection [47,48], but one RCT had a follow-up for three years [46] and an open eradication study for one year [51]. *Helicobacter* infection is linked to brady/hypokinesia cross-sectionally [52,55]. Hypokinesia lessens on the planned experiment of eradication [46,51] and increases markedly with the natural experiment of failed eradication [45,46]. With failure, the bolus release of the antigen from the killed bacteria might aggravate the immune response to the ongoing infection.

The effect of *Helicobacter* eradication may be disease modification: a differential effect was seen in IP probands (mean age 60 years) on IP facets, with less hypokinesia and more rigidity [45,46]. Reciprocal trends of worsening brady/hypokinesia and lessening rigidity subscores were seen in IP probands with untreated *Helicobacter* [51]. A pilot study [58] addressed whether the same antimicrobial intervention but for another indication would have the same outcome. Anti-*Helicobacter* therapy was given to IP probands who had antral-predominant gastritis but were biopsy-negative for *H. pylori* (rapid-urease test, histology, culture and molecular methods). No disease-modifying effect was seen.

The subsequent surveillance of all antimicrobial prescriptions in IP patients [70], with stable antiparkinsonian medication, confirmed the specificity of improved hypokinesia and bradykinesia (15 cm/year in mean stride length and 0.2 m/s/year in free walking speed) to the indication of *Helicobacter* infection. The effect on rigidity was not indication-specific: there was a cumulative increase in objectively measured rigidity following successive antimicrobial exposures for other indications (by 18% after a second course and a further 17% after a third). *Helicobacter pylori* infection might keep a rigidity-provoking source of inflammation at bay and/or eradication therapy predispose to rigidity-provoking microbiota in the small intestine and/or colon. Indeed, in IP, there is an inverse relationship between the presence of *H. pylori* and that of SIBO [71]. Moreover, an extensive dataset shows the plateauing of the year-on-year increase in objectively measured rigidity after introducing maintenance laxative [72], giving further indicative evidence for a rigidity-provoking intestinal dysbiosis.

#### 4.1.2. Consequences of Not Eradicating *Helicobacter* in IP

There is *prima facie* Level-1 OCEBM evidence on “What will happen if we do not add a therapy (eradication)?” from the current systematic review of the (Level-2) inception cohort studies [51,65], complemented by the placebo-control arm of an RCT (Level-3) [45,46]. Seemingly, there would be no change. However, the one to four years’ follow-up available is not sufficient to capture the evolution of a disease that develops over decades, with a median age at diagnosis between 60 and 69 years [73]. Since *Helicobacter* is a carcinogen, to leave it untreated (even after the endoscopic exclusion of neoplasm) for more than a year was considered inadvisable [45,46]. This, in retrospect, was a reasonable decision since nearly half were found to have pangastritis or corpus-predominant gastritis, rather than antral-predominant, and a third had atrophy or intestinal metaplasia.

In one inception cohort [65], usually with biopsy ascertainment of *Helicobacter* infection, receipt or not of eradication therapy (at mean age of 51 years) did not influence the increased risk of IP diagnosis 4 years on. Although not disease preventing, there was no evidence as to whether eradication was disease-modifying (4.1.1). In the other inception cohort [51] (mean age 63 years), with UBT ascertainment, the rigidity subscore was considerably lower one year on where *Helicobacter* was not eradicated, the change not reaching statistical significance (4.1.1). The placebo arm (mean age 63 years) in a year-long blinded-phase of an RCT of *H. pylori* eradication in IP showed no significant change in brady/hypokinesia or rigidity [46]. Recording of the sensitive markers of evolution prospectively is needed to resolve this question (e.g., higher plasma interleukin-6 concentrations were associated with a greater risk of IP 4 years on [74]).

#### 4.1.3. Inter-Relationship between *Helicobacter* and IP

Level-1 OCEBM evidence on “How common is the problem (association between *Helicobacter* and IP)?” is based on registry sample surveys, either total [63] or random, with matching (including by age and gender) of controls [65]. These embrace the concept that previous *Helicobacter* infection may be as, or more, important than current to IP aetiopathogenesis. The evidence is complemented, at Level-2, by the current and previous systematic reviews [68,69]. It is supported by non-registry surveys of IP patients, specified [52,54,64] or not specified [56,62] as consecutive, with indication that controls were matched to local circumstances [52,54,64,65]. Historical or contemporaneous *Helicobacter* is more commonly associated with IP than expected but with wide geographical variation.

Over and above different methods of ascertaining IP- and *Helicobacter*-status, the risk of the two being associated appeared considerably higher in Taiwan [65] than in Denmark [63]. This may relate to greater virulence conferred by East Asian-type *cag* pathogenicity island genes (manifest as gastric mucosal inflammatory cell infiltration; secretion of the pro-inflammatory cytokine, interleukin-8; and translocation of CagA into host cells) [75]. Even in Europe, a particular profile of antibodies against pathogenicity markers is predictive of the current disease burden and deterioration of IP over time [52]. As with gastric cancer, where a causal link to *Helicobacter* is well established, the birth cohort effect appears not to apply to IP patients: this was demonstrated with respect both to anti-urease ELISA antibody and immunoblot anti-VacA [52,62].

A 12-fold increase in all-cause mortality in IP has been associated with endoscopic biopsy positivity for an NHPH (*H. suis*) [76]. In the general population, *H. pylori* is specifically associated with gastric cancer deaths but not with all-cause mortality [77]. The magnitude of the *H. suis* effect warrants further investigation, more so in the light of the reported 10-fold greater relative frequency of *H. suis* to *H. pylori* in IP [64]. Frequency of *H. suis* was greatest in those who had received anti-*H. pylori* therapy: co-infecting *H. suis* may be filling the niche after *H. pylori* eradication. This would also fit with *H. suis* having different antimicrobial susceptibilities from *H. pylori* [78]. Human infection with NHPH is characteristically sparse and patchy, the load usually being insufficient to detect by UBT [64,76]. At present, there is one case report [58] on successful NHPH eradication in IP, with loss of antral gastritis and the associated corkscrew organisms. The patient went from wheelchair-bound and cachectic to maintaining independent mobility and weight increase during the 4-year follow-up on stable anti-parkinsonian medication. The reverse clinical picture was seen, following a third failed attempt at eradication, in an IP patient with *H. pylori-*like organisms associated with pangastritis.

### 4.2. Meeting Criteria for a Cause–Effect Relationship

When Koch (1884) formulated four criteria to establish a causative relationship between a microbe and a disease, he did not have the tools for intervention studies. Regarding (i), *The bacteria must be present in every case of the disease*, in no disease where *H. pylori* is causal is infection present in every case [79], but other *Helicobacter* species are not generally sought. Regarding (ii), *The bacteria must be isolated from the host with the disease and grown in pure culture*, isolating *H. pylori* from IP probands, and culturing it, is a positive attribute of the two RCTs in IP [45,46,47]. Regarding (iii), *The specific disease must be reproduced when a pure culture of the bacteria is inoculated into a healthy susceptible host*, IP is described only in humans, but chronic infection with *Helicobacter* might exacerbate or accelerate genetically modified animal models of hereditary parkinsonism. Regarding (iv), *The bacteria must be recoverable from the experimentally infected host*, Marshall [4,5] infected himself with *H. pylori*, producing gastritis, and subsequently eradicated it, but the latency to clinical IP may be in decades and post-date prolonged infection.

Bradford Hill (1965) [80] added, “The clear dose–response curve admits of a simple explanation”: that is causality. The discriminant index for IP, based on the anti-*H. pylori* immunoblot [52], provides an example of the quantification of pathogenicity in a dose–response relationship. Presuming that the index components are not surrogates for the pathophysiological drivers, the challenge is to understand the mechanisms.

### 4.3. Limitations and Avoiding A Priori Assumptions

#### 4.3.1. Limitations

The nature of a systematic review is to define its limits. Given that the current consensus is that IP is gut-driven and gut manifestations precede neurological diagnosis by decades [66], and immune manifestations by at least years [74], study timeframes appear to be a limitation. However, we avoid too narrow a definition of IP: it was not coherent with the above to base inclusion on a predictor of brain pathology [81] and exclude pure clinical definition [82].

The quality of the data is another limitation. Objective measurements of IP facets were used only in one eradication and two cross-sectional studies [45,46,52,55]. (e.g., Mean stride length is sensitive to the detection of small differences in the treatment effect [83] and discriminates well for IP-status, when corrected for any relevant demographic/anthropometric characteristics [42].) Indeed, in pragmatic hypothesis testing, sensitive, specific, continuous, objective measures are economical in sample size [45,46]. They avoid the carry-over effect on within-observer repetition. In contrast, using total global scores denies the possibility of selective cause/effect relationships. Moreover, effects on component subscores sensitive to intervention can be drowned out by unresponsive ones.

Inclusion of participants with levodopa-induced motor fluctuations may have obscured the effects of intervention and confounded cross-sectional comparisons. Studying the anti-IP treatment-naïve is a difficult but important option, recruiting those receiving only stable long half-time medication the next best.

Since the risk of failed *Helicobacter* eradication is considerable, false-negative conclusions may be drawn from judging the outcomes of eradication studies without protocol analysis of proven eradication. 

Low infection load of *H. pylori* appears important in IP [66] and is the norm for NHPH infection in humans. Thus, the determination of *Helicobacter-*status by endoscopic biopsy with histology, culture and molecular microbiology is ideal for research into the aetiopathogenesis of IP.

#### 4.3.2. Avoiding *A Priori* Assumptions

The assumption that any clinical improvement in IP from *Helicobacter* eradication is down to increased levodopa bioavailability has been a diversion from the basic hypothesis that *Helicobacter* is a driver in the aetiopathogenic process. The first RCT [45,46] addressed the driver hypothesis by excluding those on levodopa, but, unfortunately, most of the other cohort descriptions include levodopa usage.

We question whether *Helicobacter* infection does, indeed, influence levodopa pharmacokinetics. The levodopa concentration/time relationship is complex *per se*, including multiple peaks [53] and the inhibition of its own absorption by delaying gastric emptying [84]. Faster gastric emptying after *Helicobacter* eradication is documented both in dyspepsia alone [85] and in IP [58]. One group [47,48] provide evidence that eradication increases the area under the levodopa concentration/time curve in IP, but this is not backed up by a comparison of the infected and non-infected [53].

A second question is whether the improved performance can be ascribed to an effect of *Helicobacter* eradication on levodopa handling in the absence of conclusive evidence for altered kinetics. Elevation of baseline performance, consequent to eradicating *Helicobacter* [4.1.1], would be expected, in itself, to shorten the apparent levodopa onset time, prolong on-time and alleviate wearing-off. Moreover, it is a misconception that the increased area under the levodopa concentration/time curve equates to less severe motor fluctuations. On the contrary, increasing the effective levodopa dosage would be expected, down the line, to worsen existing fluctuations [86]. Time outside a therapeutic window of concentrations is likely to be the determinant of on/off fluctuations in performance and of dyskinesia. Pulsatile stimulation of dopaminergic receptors, consequent to faster absorption, may contribute to developing dyskinesia [87]. Inflammation alone may lower the threshold for dyskinesia and motor fluctuations. In a case report of treated NHPH infection, the onset of severe dyskinesia coincided with the return of UBT positivity, signalling recrudescence/reinfection [58]. Fluctuations are more severe where *Helicobacter* and SIBO coincide [60]: the gradual evolution of SIBO could be a determinant of delay in the onset of motor complications of levodopa. Wastage of levodopa, marked by the accumulation over time of its long t½ metabolite 3-O-methylodopa, may contribute to “wearing-off” [88].

### 4.4. Processes, Mechanisms and Explanations

Associations of IP with human leukocyte antigen-DR isotype (HLA-DR) gene loci suggest classical autoimmunity [66]. Alternatively, there could be cross-reactivity through innate pattern recognition of the *Helicobacter* genus or of a broader microbial community. In IP, the benefit of eradicating *H. pylori* on hypokinesia is independent of infection load [46]. Its persistence at a low density appears just as detrimental as at high density. All eradication failures were anti-nuclear antibody (ANA) positive, and ANA-positivity reduced the beneficial effects of “successful” eradication. Others found that autoantibodies potentially detrimental to neuronal function are associated with *H. pylori* seropositivity in IP [59].

The predicted probability of being labelled parkinsonian was greatest with anti-CagA positivity and anti-VacA and urease-B negativity [52]. This antibody profile predicted global severity, and hypokinesia and its deterioration over 4 years, within IP. There is an established link between CagA and autoimmunity in thrombocytopenic purpura [89]. Anti-CagA cross-reacts with peptides on platelets: these antibodies decline following eradication when platelet counts improve. VacA has an important role in dampening immune responses and in induction of immune tolerance early in life [90]. It shifts T-cell responses towards regulatory rather than effector functions. Thus, the absence of VacA, as in the pathogenic profile for IP, might facilitate autoimmunity driven by another antigen. Antibodies against CagA remain positive for longer than those to *H. pylori* surface antigens, such as urease B. Relying solely on anti-urease B antibodies might misclassify a significant proportion of patients who once had the infection [91].

### 4.5. Clinical and Research Implications

The inter-relationship of *Helicobacter* and IP is well-established. Eradicating *H. pylori* in IP in later adult life is disease-modifying but not disease-preventing. Eradication in childhood or young adults might reduce the risk of IP. As well as causing regression of the hypokinesia and an overall improvement in motor severity, *H. pylori* eradication appears to unlock the next stage in the natural history of IP, the development of rigidity. Already there are clues as to how rigidity can be exacerbated [70] or ameliorated [72]. Amelioration by improving intestinal transit needs to be prophylactic rather than rescue.

As a clinical consequence, screening for *H. pylori* and its eradication should be part of the routine assessment of IP. The research consequence is a building block in piecing together the aetiopathogenesis of IP. *Helicobacter pylori* virulence markers give potential mechanistic clues. The pathological impact of *Helicobacter* eradication on the gastrointestinal microbiota needs exploring in terms of both nature and site. The inverse relationship between *H. pylori* and gastric NHPH should not be ignored.

There is interest in a role for *Helicobacter* in other neuropsychiatric diseases, but IP has proven to be a good starting point since the syndrome lends itself to objective quantification and hence the definition of a pre-presentation state [35,42].

## Figures and Tables

**Table 1:** Randomised controlled trials of effect of *H. pylori* eradication on severity of, and hypokinesia in, idiopathic parkinsonism.

Study	Ascertainment of *Helicobacter* Positivity	Interventions	Number Participants (Levodopa-Status)	Primary Relevant Outcome Measure	Duration Blinded Phase (Confirmation Eradication)	Findings
Bjarnason et al. * (2005) Dobbs et al. ** (2010) [45,46]	UBT, ^†^ endoscopic biopsy (histopathology, culture and sensitivities, PCR)	1-week triple therapy, ^††^ according to in vitro sensitivities/suspected intolerance, or matched placebo (tablets/capsules without active moiety)	30: 14 active, 16 placebo (no levodopa)	Mean stride length at free walking speed	De-blinding at 54 weeks (UBT in all at 1 year, with repeat biopsy after active)	Intention to treat analysis: effect equivalent to 1.02 times between-subject SD, ^#^ whereas study designed to detect 0.75 (SD) with n = 56. ^¶^ Protocol analysis (excluding two eradication failures of blinded-active): stride length (time trend in 6-weekly measurements) improved on active by 7.3 cm/year *cf* placebo. Effect similar whether receiving stable background (long t½) anti-parkinsonian medication or treatment-naïve.
Pierantozzi et al. (2006) [47]	Serology (ELISA), stool antigen, endoscopic biopsy (rapid urease test, histopathology, culture)	1-week fixed triple therapy, ^†^ or allopurinol 100 mg twice daily for 15 days (treatments said to be balanced by placebo to equalise number pills/day and course duration)	36: 19 active 17 allopurinol (all with levodopa-induced motor fluctuations)	Sum serial UPDRS -III ^ⱡ^ scores following a levodopa dose (4 h) and including two further doses (11 h)	De-blinding at 3 months (repeat biopsy in all at 3 months	All triple therapy patients had improved scores (excluding two eradication failures), whereas, in allopurinol group, there was “lack of any significant change or even a worsening”.

* Interim analysis when recruitment stopped because of marked deterioration with failed eradication. At this stage, 31 participants had been randomised, including one drop-out with no data after blinded treatment, and 20 had reached de-blinding. ** Final analysis includes all 30 with post-treatment assessment and follow-up to 3 years after blinded-active treatment or to 2 years after open-active (following blinded-placebo). ^†^ Isotope-labelled urea breath test. ^††^ Proton pump inhibitor plus two antimicrobials. ^#^ Standard deviation. ^¶^ Sample size calculation. ^ⱡ^ Unified Parkinson’s Disease Rating Scale Part III-motor section.

**Table 2:** Non-randomised trials of effect *Helicobacter* eradication on severity of, and hypokinesia in, idiopathic parkinsonism.

Study	Ascertainment of *Helicobacter* Positivity	Interventions	Number *Helicobacter*-Positive (Levodopa-Status)	Primary Relevant Outcome Measures	Duration Follow-Up (Confirmation Eradication)	Findings
Pierantozzi et al. (2001) [48]	Anti-urease-IgG serology (ELISA)	1-week placebo followed by 1-week fixed triple therapy	6 (all with levodopa-associated wearing-off phenomenon)	UPDRS-III	2 weeks after each intervention (none)	UPDRS score lower after triple therapy than after placebo at 2 h after levodopa test-dose but not 1 h.
Lee et al. (2008) [49]	UBT	1-week fixed triple therapy	35 (all with levodopa-induced motor fluctuations)	UPDRS-III	3 months (UBT at 9 weeks)	Numerical, but not statistically significant, reduction in UPDRS after eradication (excluding one eradication failure).
Hashim et al. (2014) [50]	UBT	1-week fixed triple therapy	21 * (levodopa therapy for ≥1 month)	UPDRS-III PDQ-39 ^†^	12 weeks (UBT at 12 weeks)	UPDRS and PDQ scores (including mobility) improved significantly (by 13 and 19 points, respectively) after eradication.
Liu et al. (2017) [51]	UBT	2-week fixed triple therapy	24 (half untreated) (levodopa used but not inclusion criterion)	UPDRS-III	1 year (UBT at 1 year)	Within triple therapy patients, and by comparison with untreated, improvement in UPDRS score, mainly brady/hypokinesia subscores (excluding two eradication failures).

* After excluding six drop-outs. ^†^ Parkinson’s Disease Questionnaire (disability).

**Table 3:** Cross-sectional observational studies of relationship of *Helicobacter*-status to motor severity and hypokinesia within idiopathic parkinsonism.

Study	Ascertainment of *Helicobacter* Positivity	Number of IP * Participant(s) (Levodopa-Status)	Primary Relevant Outcome Measures	Findings
Weller et al. (2005) [52]	Serum immunoblot antibody profile	124 with immunoblot profile (81% on levodopa, evenly spaced to minimise fluctuations)	Stride length and its deterioration over 4 years Webster Rating Scale	Gradients of clinically relevant size between “discriminant index for IP-status” (based on immunoblot) and shorter stride length and more deterioration over 4 years. Gradient between index and Webster rating.
Lee et al. (2008) [49]	UBT	30 *Helicobacter*-negative, 35 positive (all on levodopa)	UPDRS-III ^†^	No difference in UPDRS.
Hashim et al. (2014) [50]	UBT	53 *Helicobacter*-negative, 21 positive (all on levodopa for ≥1 month)	UPDRS-II and -III PDQ-39 ^†^	*Helicobacter*-positives had worse UPDRS and PDQ scores.
Narozanska et al. (2014) [53]	Stool antigen	48 *Helicobacter*-negative, 25 positive (all with levodopa-induced motor fluctuations)	UPDRS-III ^†^	No difference between *Helicobacter*-positives and -negatives in UPDRS.
Bu et al. (2005) [54]	Anti-urease-IgG serology (ELISA)	131 with antibody-status against six pathogens (including *H. pylori*), compared with 141 without IP (unspecified number on levodopa)	Schwab and England Stage ^†^	Infection burden, based on number seropositivities for six pathogens, associated with having IP and its Schwab and England Stage.
Tan et al. (2015) [55]	UBT	69 *Helicobacter*-negative, 33 positive (unspecified number on levodopa)	UPDRS-III Timed walking test Purdue Pegboard test	*Helicobacter*-positive had worse UPDRS, longer timed waking test and inserted fewer pegs in allotted time.
Esmael et al. (2016) [56]	Anti-urease-IgG serology (ELISA)	27 *Helicobacter*-negative, 23-positive (all with levodopa-induced motor fluctuations)	Total UPDRS (six parts) PDQ-39 ^†^	UPDRS and PDQ worse in *Helicobacter*-positive.
Roshan et al. (2018) [57]	Anti-urease-IgG serology (ELISA)	66 *Helicobacter*-negative, 33-positive (no information on levodopa receipt)	UPDRS-II & -III ^†^	No difference between *Helicobacter*-positive and -negative in UPDRS.

* idiopathic parkinsonism. ^†^ Hoehn & Yahr Stage also performed but no difference found in Stage according to *Helicobacter*-status.

**Table 4:** Cross-sectional observational studies of relationship of *Helicobacter*-status to other outcomes in idiopathic parkinsonism.

Study	Ascertainment of *Helicobacter-*Positivity	Number Participants (Levodopa-Status)	Primary Relevant Outcome Measure	Findings
Dobbs et al. (2005) [58]	Serum immunoblot antibody profile	124 with IP and 194 without IP (81% on levodopa, evenly spaced to minimise fluctuations)	Body mass index	Odds of being underweight tripled in presence of anti-VacA ^†^ antibodies, irrespective of IP-status.
Suwarnalata et al. (2016) [59]	Serology (ELISA), anti-CagA, ^††^ anti-*H. pylori* whole cell	60 with IP, of which 30 seropositive (unspecified number on levodopa)	Autoantibody screen	13 autoantibodies “against proteins essential for normal neurological functions” discriminated *Helicobacter*-positive from -negative.
Fasano et al. (2013) [60]	UBT	33 with IP of which 11 UBT-positive and 18 hydrogen breath test positive for SIBO ^#^ (all with levodopa-induced motor fluctuations)	Levodopa-induced motor complications	Unpredictable fluctuations were significantly more with SIBO plus *Helicobacter*-positivity, and tended to be more with SIBO alone, than in absence of both.
Rahne et al. (2013) [61]	UBT	40 with IP of which 20 UBT-positive (all taking levodopa)	Levodopa-induced motor complications	*Helicobacter*-positives had worse UPDRS-IV.

^†^ anti-vacuolating toxin. ^††^ anti-cytotoxin-associated gene product. ^#^ SIBO = small intestinal bacterial overgrowth.

**Table 5:** Observational studies of comparative frequency of associated *Helicobacter* and idiopathic parkinsonism and its age relationship.

Study	Ascertainment of *Helicobacter* Positivity	Country	Number Participants or Samples	Participant/Sample Source	Findings
Dobbs et al. (2000) [62]	Anti-urease-IgG serology (ELISA)	UK	105 with IP, 210 controls	Call for volunteers with and without IP followed by screening for inclusion/exclusion criteria.	Controls showed birth cohort effect. Having IP obliterated this. Higher frequency of *Helicobacter*-positive, up to 72.5 years, in IP than in controls.
Weller et al. (2005) [52]	Immunoblot serology Anti-urease-IgG serology (ELISA)	UK	124 with IP, 196 without	Consecutive IP patients attending clinic and consecutive responders to call for healthy volunteers from same locality. Similar inclusion/exclusion criteria applied to both, except diagnosed IP an exclusion in controls.	Controls showed a birth cohort effect for odds of having VacA antibody. Having IP obliterated this. Predicted probability of IP greatest with CagA-positivity, VacA-negativity and urease-B negativity. Discrimination not complemented by ELISA.
Nielsen et al. (2012) [63]	*Helicobacter* eradication course	Denmark	4484 with IP, 22416 controls	Danish National Patient Register for diagnosis IP, dated to first prescription IP drug. National Prescription Registry. Index date for IP diagnosis between 2001 and 2008, eradication date from 1995. Danish Civil Registration System source of 5 matched controls per IP patient.	Frequency of historical *Helicobacter* eradication increased in IP, even when only eradications ≥5 years prior to IP assessed.
Blaecher et al. (2013) [64]	For *H. pylori*: culture and PCR in culture negative For *H. suis*: PCR.	UK	60 DNA extracts from people with IP, 256 DNA extracts from people attending endoscopy departments	Serial archived DNA extracts from *Helicobacter* Reference Laboratory, Public Health England, with selection for adequate documentation and whether from IP clinic or not.	Relative risk of *H. suis* compared with *H. pylori* 10 times greater in IP than in controls.
Fasano et al. (2013) [60]	UBT	Italy	33 with IP, 30 spouses of IP probands	Consecutive IP patients attending a hospital and their spouses.	Similar frequency of *Helicobacter* positivity in IP and spouses.
Bu et al. (2005) [54]	Anti-urease-IgG serology (ELISA)	China	131 IP, 141 controls	Consecutive IP patients attending a hospital. Controls randomly recruited from hospital’s clinics over same period.	Higher frequency of *Helicobacter* positivity in IP.
Esmael et al. (2016) [56]	Anti-urease-IgG serology (ELISA)	Egypt	50 with IP, 20 controls	IP patients from neurology outpatients. Age and gender matched controls with no diagnosed neurological disease.	Higher frequency of *Helicobacter* positivity in IP.
Huang et al. (2018) [65]	Endoscopic-biopsy, with UBT where endoscopy not tolerated	Taiwan	9186 *Helicobacter*-positive (reduced to 9105 after matching), 9105 *Helicobacter*-negative	“2000 Longitudinal Health Insurance Database”. Propensity score matching based on age, sex, income, urbanization, comorbidities, medication). Index date 2000 to 2011 inclusive. Follow-up until IP diagnosis, death or latest 2012.	Increased estimated incidence IP in *Helicobacter*-positive ≥60 years, irrespective of eradication therapy.
Roshan et al. (2018) [57]	Anti-urease-IgG serology (ELISA)	Iran	99 with IP, 297 controls Exclusion if history of *Helicobacter* eradication	Consecutive IP patients in neurology clinic. “Amirkola Health and Ageing Project”: three controls, with no history of IP drugs, matched to each IP proband for age and gender.	Lower frequency of *Helicobacter* positivity in IP.

**Table 6:** Risk of bias assessment of randomised placebo-controlled trials.

Citation	Selection Bias		Performance Bias	Detection Bias	Attrition Bias	Reporting Bias
	Random Sequence Generation	Allocation Concealment	Blinding of Participants & Personnel	Blinding of Outcome Assessment	Incomplete Outcome Data	Selective Reporting
[45,46]	low	low	low	low	low	low
[47]	low	unclear *	low	moderate †	low	unclear #

* Patients allocated to either 7 days of twice daily *Helicobacter* eradication triple-therapy or 15 days of a twice daily allopurinol pill. All participants “received the same number of tablets”, but 7 (rather than 8) days of placebo specified at end of triple therapy. † “An expert neurologist unaware of study design clinically evaluated patients.” This involved serial within-day and between-day subjective scoring. # Whether intention-to-treat or protocol analysis of proven eradication.
